# Supplementary material for: rhTPO Ameliorates Radiation-Induced Long-Term Hematopoietic Stem Cell Injury in Mice
Source: Molecules. 2023 Feb 18;28(4):1953. doi: 10.3390/molecules28041953 (PMC9961369; doi:10.3390/molecules28041953)
Supplement: Supplementary file 1 [file molecules-28-01953-s001.zip › molecules-2174091-supplementary.pdf]

Supplementary Materials

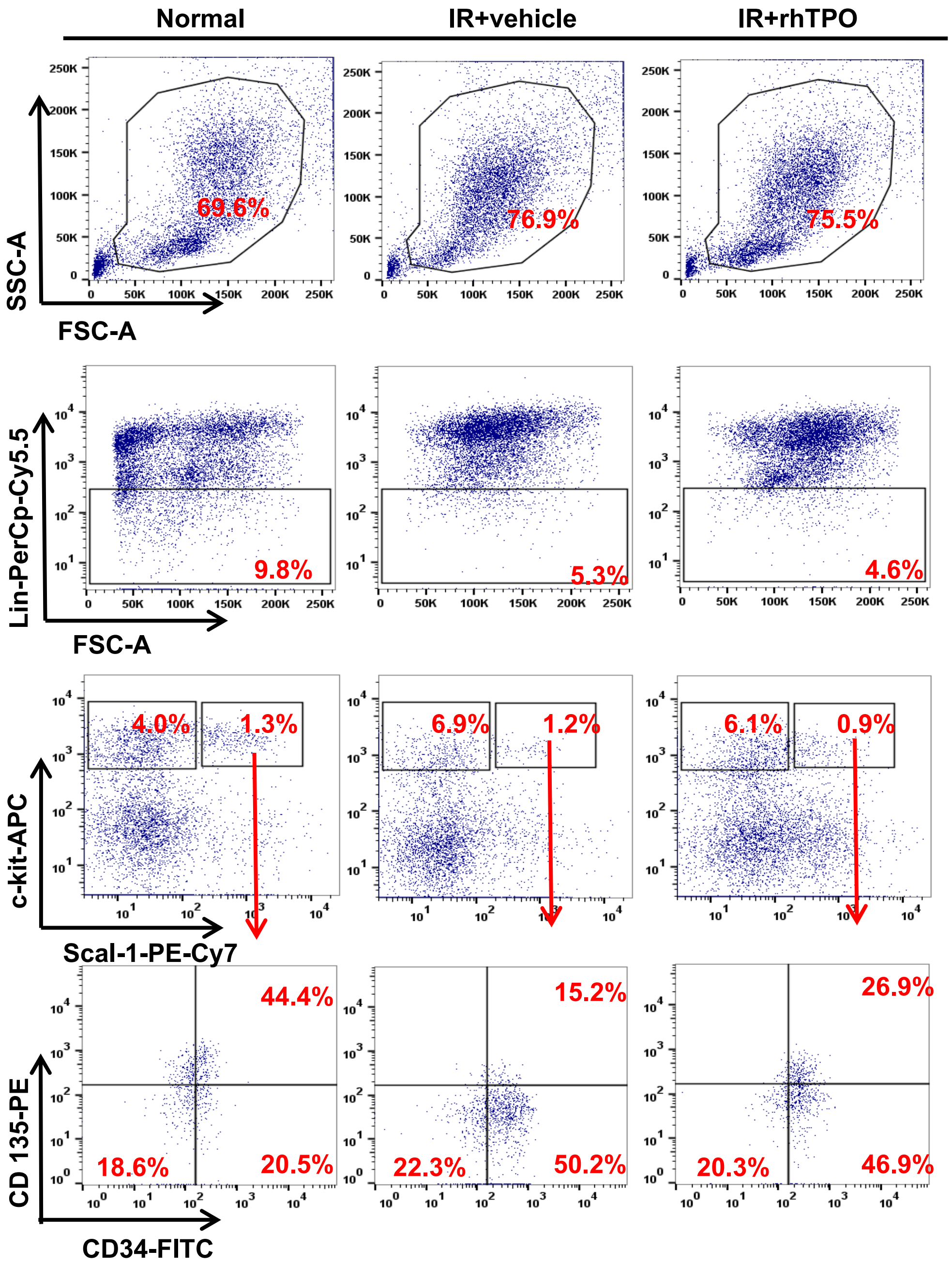

Supplementary Figure S1: Representative FACS plots for HSC (Lin−Scal+c-kit+), HPC (Lin−Scal−c-kit+), LT-HSC (CD34−CD135−LSK), ST-HSC (CD34+CD135−LSK), and MPP (CD34+CD135+LSK) cell populations.
